# Percutaneous coronary intervention in asians- are there differences in clinical outcome?

**DOI:** 10.1186/1471-2261-11-22

**Published:** 2011-05-23

**Authors:** Angela S Koh, Lay W Khin, Lok M Choi, Ling L Sim, Terrance S Chua, Tian H Koh, Jack W Tan, Stanley Chia

**Affiliations:** 1 National Heart Centre Singapore, Singapore

## Abstract

**Background:**

Ethnic differences in clinical outcome after percutaneous coronary intervention (PCI) have been reported. Data within different Asian subpopulations is scarce. We aim to explore the differences in clinical profile and outcome between Chinese, Malay and Indian Asian patients who undergo PCI for coronary artery disease (CAD).

**Methods:**

A prospective registry of consecutive patients undergoing PCI from January 2002 to December 2007 at a tertiary care center was analyzed. Primary endpoint was major adverse cardiovascular events (MACE) of myocardial infarction (MI), repeat revascularization and all-cause death at six months.

**Results:**

7889 patients underwent PCI; 7544 (96%) patients completed follow-up and were included in the analysis (79% males with mean age of 59 years ± 11). There were 5130 (68%) Chinese, 1056 (14%) Malays and 1001 (13.3%) Indian patients. The remaining 357 (4.7%) patients from other minority ethnic groups were excluded from the analysis. The primary end-point occurred in 684 (9.1%) patients at six months. Indians had the highest rates of six month MACE compared to Chinese and Malays (Indians 12% vs. Chinese 8.2% vs. Malays 10.7%; OR 1.55 95%CI 1.24-1.93, p < 0.001). This was contributed by increased rates of MI (Indians 1.9% vs. Chinese 0.9% vs. Malays 1.3%; OR 4.49 95%CI 1.91-10.56 p = 0.001), repeat revascularization (Indians 6.5% vs. Chinese 4.1% vs. Malays 5.1%; OR 1.64 95%CI 1.22-2.21 p = 0.0012) and death (Indians 11.4% vs. Chinese 7.6% vs. Malays 9.9%; OR 1.65 95%CI 1.23-2.20 p = 0.001) amongst Indian patients.

**Conclusion:**

These data indicate that ethnic variations in clinical outcome exist following PCI. In particular, Indian patients have higher six month event rates compared to Chinese and Malays. Future studies are warranted to elucidate the underlying mechanisms behind these variations.

## Background

The widespread utilization of PCI as a treatment strategy for coronary artery disease has altered the management of patients with CAD [1,2]. Several reports have examined the outcomes following PCI of various patient populations [3-9]. While population registries a decade ago have shown differences in coronary mortality following an acute myocardial infarction (MI) among various ethnic groups, contemporary reports of differences in clinical outcomes following PCI among Asians remain limited [10-13].

The objective of this study was to examine, in a contemporary group of patients from various Asian subpopulations in a similar social environment, the impact of ethnic differences on clinical outcome after PCI for CAD.

## Methods

### Design and study population

We designed a prospective registry of all consecutive patients who were referred for PCI between January 2002 and December 2007, including both emergency as well as elective PCI procedures. Ethical approval for the study was obtained from our local institutional review board and patient consent obtained where appropriate.

The ethnic group of the patient was obtained from the source of notification, based on the national census classification. We included the three major ethnic groups comprising of Chinese, Malays and Indians, and excluded the other remaining minority groups from the analysis.

Baseline clinical characteristics were collected during the procedure. Follow-up data was obtained from medical records or telephone follow-up. The primary endpoint of this analysis was major adverse cardiac event (MACE) rates defined as nonfatal MI, repeat revascularization and all-cause death at six months post-index PCI. Secondary endpoints were one month MACE, and individual MACE components at one month and six months.

If a patient underwent more than one PCI during this time frame, the initial one was taken as the index procedure.

### Statistical analysis

Categorical variables were presented as percentages and compared using chi-square/Fisher's exact test. Continuous variables are presented as the mean ± standard deviation and comparisons were made by means of analysis of variance. Univariate analysis was performed to compare demographic and baseline characteristics among the ethnic groups. Stepwise multivariate logistic regressions were carried out to assess the association between one- and six-months adverse events for Malay and Indian patients as compared to Chinese patients with correction for significant differences in baseline diabetes, family history of CAD, tobacco smoking and primary PCI (Table 1). Statistical significance was assumed if p < 0.05.

**Table 1:** Baseline Clinical Characteristics

Variable	Chinese	Malay	Indian	p value
**No. of patients (n, %)**	5130 (68)	1056 (14)	1001 (13.3)	
**Age (mean ± SD, years)**	60.0 ± 10.7	56.8 ± 10.7	56.9 ± 10.6	0.614
**Male n (%)**	4016 (78.6%)	854 (78.0%)	793 (79.2%)	0.887
**Hypertension**	3561 (69.7%)	727 (66.4%)	659 (65.8%)	0.312
**Dyslipidemia**	4012 (78.5%)	866 (79.1%)	817 (81.6%)	0.150
**Diabetes**	1702 (33.3%)	491 (44.8%)	484 (48.4%)	<0.0001
**Family History of CAD**	540 (10.6%)	125 (11.4%)	191 (19.1%)	<0.0001
**History of tobacco smoking**	2339 (45.8%)	599 (54.7%)	436 (43.6%)	<0.0001
**Stable angina**	1955 (38.3%)	387 (35.3%)	349 (34.9%)	0.432
**Unstable angina**	1147 (22.4%)	205 (18.7%)	217 (21.7%)	0.066
**Primary PCI**	573 (11.2%)	128 (11.7%)	148 (14.8%)	0.002

Baseline clinical characteristics of the analyzed population.

MI: myocardial infarction; PCI: percutaneous coronary intervention.

Values are given as number of cases (%). Continuous variable (e.g., age) is expressed as mean ± standard deviation (SD).

Data management and analysis were performed using the Statistical Package for Social Sciences, (SPSS, Chicago Inc.) Windows version 16.

## Results

A total of 7889 consecutive patients who underwent PCI between January 2002 and December 2007 were enrolled in our registry, and 7544 (96%) patients who completed follow-up were included in this present analysis. In this patient group (n = 7544), 68% (n = 5130) were Chinese, 14% (n = 1056) were Malay, 13.3% (n = 1001) were Indian, and 4.7% (n = 357) were patients from other minority ethnic groups.

Compared with Chinese patients, there was a higher incidence of diabetes mellitus, family history of CAD and history of tobacco smoking among Indian and Malay patients (Table 1). Apart from a higher incidence of primary PCI for ST-segment elevation myocardial infarction among Indians, the clinical indications for referral for PCI were similar among the ethnic groups. Bare metal stents were used in 56% of the entire cohort while drug-eluting stents were used in the remaining 44%; there were no differences in the type of stent used among the difference ethnic groups. Compliance to dual antiplatelet therapy from time of PCI to hospital discharge was satisfactory; 93.5% of patients were discharged with Aspirin while 93.9% of patients were discharged with clopidogrel.

Comparison of the primary endpoint of any major adverse cardiovascular event between ethnic groups is shown in Table 2.

**Table 2:** Primary and Secondary Outcomes among Ethnic Groups

	Chinese	Malay	Indian	p value
**Any MACE**				
One month	209 (4.1%)	57 (5.2%)	66 (6.6%)	0.004
Six months	418 (8.2%)	117 (10.7%)	120 (12%)	<0.0001
**Revascularization**				
One month	193 (3.8%)	53 (4.8%)	62 (6.2%)	0.003
Six months	207 (4.1%)	56 (5.1%)	65 (6.5%)	0.005
**Death**				
One month	15 (0.3%)	3 (0.3%)	4 (0.4%)	0.95
Six months	387 (7.6%)	108 (9.9%)	114 (11.4%)	<0.0001
**MI**				
One month	12 (0.2%)	4 (0.4%)	10 (1.0%)	<0.001
Six months	44 (0.9%)	14 (1.3%)	19 (1.9%)	0.029

Individual components of one month and six-month MACE in the overall cohort.

Values are given as number of cases (%).

**Figure 1:**
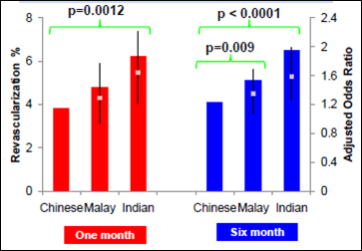
**Adjusted one month and six-Month rates of revascularization which adjusts for all variables in Table 1**.

**Figure 2:**
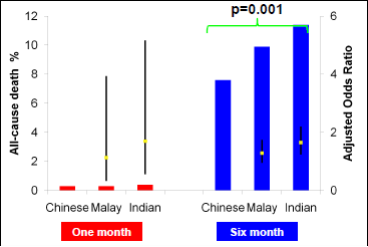
**Adjusted one month and six-Month rates of death which adjusts for all variables in Table 1**.

At six months, the primary endpoint occurred more frequently among Indian and Malay patients than Chinese patients [12% vs. 10.7% vs. 8.2% respectively at six months (p < 0.001)]. Compared to Chinese patients, Malays also had a higher likelihood of repeat revascularization (OR 1.35 95%CI 1.08-1.69, p = 0.009) and MACE at six months (OR1.36 95%CI 1.09-1.69, p = 0.006) (Figure 1 and Table 2). Indian patients had higher risks of mortality (OR1.65 95%CI1.23-2.20 p = 0.001) (Figure 2) and required more revascularization procedures (OR1.59 95%CI 1.27 1.98 p < 0.001) compared to the Chinese (Figure 1).

One-month outcomes were significantly worse among Indian patients compared to Chinese patients. Indians and Malays were more likely than Chinese patients to have higher MACE rates at one month [6.6% vs. 5.2% vs. 4.1% respectively (p = 0.004)].

**Figure 3:**
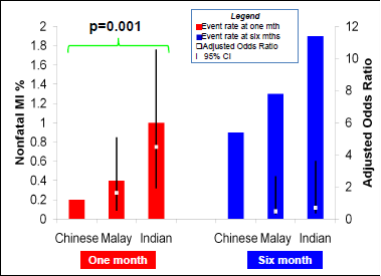
**Adjusted one month and six-Month rates of myocardial infarction which adjusts for all variables in Table 1**.

Myocardial infarction was seen in 1.0% versus 0.2% (OR4.49 95%CI 1.91-10.56 p = 0.001) (Figure 3) in Indian versus Chinese patients and overall MACE was 6.6% versus 4.1% (OR1.64 95%CI 1.23-2.19 p = 0.001). The frequency of revascularization procedures during one-month follow-up also differed by ethnicity (Figure 1). Compared to Chinese patients, Malay and Indian patients were significantly more likely to require revascularization procedures at one-month (3.8% vs. 4.8% vs. 6.2% respectively, p = 0.0012).

## Discussion

Our results demonstrate higher risks of adverse cardiovascular outcome among Indian and Malay patients, compared to Chinese patients, after PCI. This was largely due to higher rates of myocardial infarction, death and repeat revascularization among Indians and higher rates of repeat revascularization among Malays.

To the best of our knowledge, this is the first large study to report on the differences in outcomes among Asian patients.

We have found that Indian patients who were referred for PCI have more cardiovascular risk factors at baseline than Chinese patients. Indians had higher rates of diabetes mellitus and family history of coronary artery disease compared to Chinese. Indian patients also presented more often with acute ST-segment elevation myocardial infarction that required primary PCI. This was associated with higher rates of short-term major adverse cardiovascular events among Indian patients, driven by higher rates of myocardial infarction and need for repeat revascularization procedures after one-month.

The higher rates of mortality up to six months among Indians compared to Chinese concur with previous reports of excess mortality from ischemic heart disease amongst Indians [10,12]. This finding is similar to earlier reports of South Asian patients living in other parts of the world who experience higher risk of death over the ensuing six months than the local population [14,15].

Given the considerably higher rates of MACE among Indian and Malay patients in our population, it is important to identify potential causes for these findings [11,16]. It has been reported that Asian Indians are more susceptible to the development of diabetes mellitus than Chinese and Malays [15-18] that may account for worse outcomes. However, we have adjusted for these differences in risk factors and the resulting outcomes are still much worse amongst Indians and Malays.

Our study has several limitations. Firstly, this study is based on registry data and only included patients who were referred for PCI for CAD. This would have excluded patients with underlying CAD who were not referred for PCI or did not require PCI due to either physician or patient choice. The resulting clinical outcomes of these patients would be quite different due to referral bias.

Secondly, the endpoints were not independently adjudicated. However, this is study represents a significant sample of a multi-ethnic population for which the primary and secondary outcomes were reviewed carefully from medical records and telephone follow-up over time.

Given these findings, it is likely that there are other yet unknown mechanisms that may account for the disparity in clinical outcomes amongst the three major ethnic groups. It is unlikely that these differences can be explained on the basis of co-morbidities alone. It would be important in future studies to explore the role of other treatment factors like compliance, racial differences in rates of disease progression or response to drug therapy on cardiovascular outcomes in these Asian subpopulations.

## Conclusions

Despite advances in CAD management, differences in racial outcomes after percutaneous coronary intervention are evident in our study. The larger burden of cardiovascular disease risk factors among the ethnic groups may not completely explain these differences. To fully understand this ethnic inequality in cardiovascular outcomes, we will require further research in order to elucidate the reasons that underlie these differences and work towards reducing these disparities.

## Competing interests

The authors declare that they have no competing interests.

## Authors' contributions

A S. Koh is the first author who analyzed and interpreted data, drafted and completed the manuscript. L W. Khin made substantial contributions to statistical analysis. L M Choi participated in conception, design and acquisition of the data. L L. Sim participated in conception, design and acquisition of the data. T S. Chua was involved in critical appraisal of the manuscript. T H. Koh was involved in the coordination and acquisition of data. J W. Tan was participated in the design and interpretation of data. S. Chia conceived and designed the manuscript, analyzed and interpreted the data, involved in drafting of the manuscript and revising it for content.

All authors have read and approved the final manuscript.

## Pre-publication history

The pre-publication history for this paper can be accessed here:

http://www.biomedcentral.com/1471-2261/11/22/prepub
